# New global targets for NTDs in the WHO roadmap 2021–2030

**DOI:** 10.1371/journal.pntd.0009373

**Published:** 2021-05-13

**Authors:** Adriano Casulli

**Affiliations:** 1 European Union Reference Laboratory for Parasites, Department of Infectious Diseases, Istituto Superiore di Sanità, Rome, Italy; 2 WHO Collaborating Centre for the Epidemiology, Detection and Control of Cystic and Alveolar Echinococcosis, Department of Infectious Diseases, Istituto Superiore di Sanità, Rome, Italy; George Washington University School of Medicine and Health Sciences, UNITED STATES

## Abstract

The second World Neglected Tropical Diseases (NTDs) Day was celebrated on 30 January 2021. To mark the occasion, the World Health Organization (WHO) launched its roadmap for NTDs for the period 2021 to 2030, which is aimed at increasing prevention and control of these too-long neglected diseases. Described here is a global overview on past achievements, current challenges, and future prospects for the WHO NTDs roadmap 2021–2030.

## What are NTDs and where can be found

According to the WHO criteria for classification, NTDs are diseases, disorders, or conditions that (1) disproportionately affect poor and marginalized populations, causing important morbidity and mortality, therefore justifying a global response; (2) mainly affect, but are not limited to, communities living in tropical and subtropical areas, especially those far from healthcare settings; (3) can be prevented and controlled by public health interventions; and (4) are relatively neglected by scientific research and public/private funding, compared to the magnitude of the health problem [1].

Based on the above criteria, WHO currently focuses on a diverse group of 20 diseases and disease groups, mainly infectious, caused by (lyssa- and arbo-)virus, bacteria, fungi, parasites (protozoa and helminths), and toxins (snake bite envenoming, noncommunicable disease), all of global public health importance (**Box 1**). This priority list does not account for all the neglected clinical conditions causing health, social, and economic burdens worldwide. For instance, *PLOS Neglected Tropical Diseases* has significantly expanded this list to include additional diseases or conditions with chronic and/or debilitating characteristics comparable to the core NTDs group [2].

Box 1. Alphabetical list of currently endorsed NTDs in the portfolio of WHO *[synonymous] (forms of the same disease or diseases caused by different etiologic agents)

- Buruli ulcer
- Chagas disease [American trypanosomiasis]
- Dengue and Chikungunya
- Dracunculiasis [Guinea-worm disease]
- Echinococcosis (alveolar, cystic, neotropical)
- Foodborne trematode infections (clonorchiasis, opisthorchiasis, fascioliasis, paragonimiasis)
- Human African trypanosomiasis, HAT [sleeping sickness] (gambiense and rhodesiense forms)
- Leishmaniasis (cutaneous, mucocutaneous, visceral)
- Leprosy [Hansen disease]
- Lymphatic filariasis [Elephantiasis]
- Mycetoma, chromoblastomycosis, and other deep mycoses
- Onchocerciasis [river blindness]
- Rabies
- Scabies and other ectoparasitoses
- Schistosomiasis [bilharzia] (intestinal and urogenital)
- Soil-transmitted helminthiases, STHs (ascariasis, ancylostomiasis, necatoriasis, trichuriasis, strongyloidiasis)
- Snake bite envenoming
- Taeniasis and cysticercosis
- Trachoma
- Yaws (Endemic treponematoses)

*Adapted from https://www.who.int/teams/control-of-neglected-tropical-diseases

Irrespective of classifications, the vast majority of NTDs are prevalent in tropical and subtropical regions of Africa, America, Asia, and Oceania. However, some of them historically extend beyond these borders. For example, leishmaniasis, cystic echinococcosis, and alveolar echinococcosis are historically endemic in Europe [3,4]. The observed recrudescence of NTDs’ presence out of core endemic areas can be attributed to global societal and climatic changes. Events such as human migration, international travel, animal movements and trade, food trade, economic downturns, and climate changes may extend the areas of presence of pathogens causing NTDs, their mammalian hosts, the transmission season, and competence of vectors, spreading NTDs worldwide. This is the case of some poverty-related protozoan and helminthic NTDs in southern United States and vector-borne NTDs and schistosomiasis in southern Europe [5–7]. In the case of other arboviral NTDs, such as dengue and chikungunya, an autochthonous transmission cycle has now been established in southern Europe [8].

## Main challenges, successes, and failures during last two decades

An alternative, pragmatic approach is to list NTDs on the basis of progresses and failures towards goals set for their prevention, control, elimination, and eradication. A major part of the success in reducing the health, social, and economic burden of NTDs can be attributed to the historical implementation of integrated programmes of mass drug administration (MDA, also referred to as preventive chemotherapy) focusing on single use or combination of mainly 5 drugs (albendazole or mebendazole, ivermectin, praziquantel, and azithromycin) targeting major soil-transmitted helminthiases (STHs; ascariasis, trichuriasis, ancylostomiasis, necatoriasis), lymphatic filariasis, onchocerciasis, schistosomiasis, and trachoma. More recently, it has been noted that MDA might have collateral benefits on other conditions such as strongyloidiasis, scabies, loiasis, mansonelliasis, foodborne trematodiases, taeniasis, and yaws [9]. Since 2011, over 20 billion doses of quality-assured medicines for MDA were donated by the pharmaceutical companies to endemic countries to support control and elimination of NTDs, and more than 1 billion people/year have been treated for at least one disease for 5 consecutive years (2015 to 2019) [10]. Only in 2019, 2.7 billion treatments by means of MDA were delivered for NTDs [10]. In this context, WHO estimated that 500 million fewer people were in need of interventions in 2019 compared to 2010, with 42 countries having eliminated at least one of the 20 prioritized NTDs [11]. Thanks to scale-up of interventions and progress made during the last decades, there are now 5 NTDs whose eradication, elimination, or elimination as a public health problem is on the horizon: dracunculiasis, gambiense form of Human African Trypanosomiasis (HAT), lymphatic filariasis, trachoma, and yaws.

Dracunculiasis is, in theory, not far from eradication, with only 54 human cases reported in 4 countries in 2019 and 24 cases reported until end of November 2020 [12]. However, the recent finding of a genetically identical Guinea worm population, which infect both human and dogs, seems to rise complications in this public health effort [13].

During 2012 to 2019, thanks to improved surveillance, case detection, treatment, and vector control, the annual number of human HAT (mainly the gambiense form) has dropped from over 7,000 cases to 980. A prevalence reduction of 74% of lymphatic filariasis was achieved during the period 2000 to 2018 [14], thanks to support by the Global Programme to Eliminate Lymphatic Filariasis (GPELF). Lymphatic filariasis has now been eliminated as a public health problem in 17 countries and trachoma in 10 countries [11]. Between 2002 and 2020, the implementation of the SAFE strategy for trachoma has decreased by 91% the population requiring antibiotics, facial cleanliness, and environmental improvement for this disease [15]. For yaws, a new eradication strategy based on community interventions, also known as Morges strategy, was launched in 2012 after the finding that a single dose of oral azithromycin is at least as efficacious as intramuscular penicillin in achieving cure [16,17].

Onchocerciasis has further reduced its burden during the period 2005 to 2017 under the African Programme for Onchocerciasis Control (APOC). In the Americas, transmission interruption was verified by WHO in 4 Latin American countries, with low levels of transmission persisting in Brazil and Venezuela [18,19].

The main goal for the control of the 2 major forms of schistosomiasis (intestinal and urogenital) is to reach by means of praziquantel MDA, more than 75% treatment coverage of school-aged children, at-risk adults, and communities living in highly endemic areas [20]. In 2019, 77.8 million people (64.9 million school-aged children and 12.9 million adults) were treated by MDA for schistosomiasis, corresponding to 34.6% of those in need [21].

STHs are among the most common NTDs. Up to now, more than 3.3 billion benzimidazole tablets (albendazole or mebendazole) have been distributed to school-aged children, reaching a coverage level of 60% between 2008 and 2018 [22] and averting over 40% of the disability-adjusted life years (DALYs) lost annually in children in 2015 [23]. MDA integrated campaigns with albendazole and mebendazole were successful in reducing disease prevalence of ascariasis while less effective against hookworm infections (ancylostomiasis, necatoriasis) and trichuriasis. In principle, such low efficacy can be partly overcome by combining either oxantel pamoate or ivermectin [24].

Although human deaths associated to dog-mediated rabies (95% of all-deaths from rabies) are estimated at around 23,500 to 59,000 per year, mostly children younger than 15 years living in Asia and Africa, elimination was achieved in Canada, Western Europe (in 2019, only Poland and Romania were reporting cases in European Union), USA, Japan, and substantial decrease in Latin American countries [25–27]. The highly committed “Zero by 30” global strategic plan to eliminate dog-mediated rabies is now in place, with the aim of guiding effective use of vaccines, medicines, and technologies, and generating both evidence-based guidance and high-quality data for control [28]. Since 2010, total number of new leprosy cases declined by 27% after most endemic countries reached its elimination (defined as a prevalence rate of <1 case on treatment /10,000 population) as a public health [29].

Since 2012, the number of reported cases of visceral leishmaniasis has slightly decreased globally and fallen significantly in Bangladesh, India, and Nepal, where the disease is targeted for elimination as a public health problem [30]. A better access to diagnosis and treatment, coupled with aggressive vector control strategies and elimination initiative in Asia, has led to these results; nevertheless, human conflicts and increased competence of vectors due to global warming have seen the rise of leishmaniasis in many areas, particularly the Middle East and East Africa [30].

During the last decade, the need for surgery for Buruli ulcer (mainstay of treatment before 2005) has declined by 50%, thanks to the availability of an 8-week antibiotic combination therapy [31]. More recently, an open-label Phase III randomized trial supported by WHO demonstrated that Buruli ulcer is curable with an 8-week course of oral rifampicin plus extended-release clarithromycin, the latter drug replacing intramuscular streptomycin that is painful and potentially ototoxic; in addition, surgery is not required in these patients [32].

Sensible but less effective gains were obtained for some other NTDs, globally or in some specific geographic areas such as Latin America or Asia-Pacific region. Although vector control efforts progress in the Southern Cone of South America, challenges remain in the global fight against Chagas disease, which still affects around 6 to 8 million people worldwide, the vast majority in Latin America but also expanding in southern USA and Europe, mainly as nonvectorial transmission. Only 2 medicines are currently available for the treatment of Chagas disease (benznidazole and nifurtimox), both of which present serious side effects; in addition, their efficacy has been proved only during the early acute phase of infection, while benefits in the chronic phase are questionable. In fact, the BENEFIT randomized study demonstrated that benznidazole administered after the onset of chronic Chagas cardiomyopathy does not alter the progression of heart disease, neither decrease mortality rate [33]. New drug association, drug repositioning, research of new drug, and therapeutic vaccine are under evaluation for Chagas disease [34,35].

When comparing Global Burden of Disease (GBD) data from 2000 to 2017, NTDs such as foodborne trematode infections, dengue, and echinococcosis are increasing in Asia-Pacific region by 21%, 109%, and 59%, respectively [36]. Such huge insurgence of dengue in Asia is not unexpected since this arbovirus infection is rising worldwide due to the increase of urbanization in the tropics, adaptation of the main vector to urban environments, and climate changes [37]. Similarly, zoonotic cestodes such as taeniasis/cysticercosis, cystic echinococcosis, and alveolar echinococcosis are considered the first, second, and third most relevant foodborne parasitic diseases at the global level [38]. Irrespective of that, these neglected helminths are losing ground, since a limited portfolio of antiparasitic drugs is available, while less and not systematic efforts have been globally sustained during the past years. MDA campaigns with praziquantel were previously conducted against taeniasis/cysticercosis in Latin American countries (Ecuador, Guatemala, Mexico, Peru), Asia (Lao People’s Democratic Republic, Nepal), and Africa (Tanzania and Madagascar) [39]. More recently, a three-phase large eradication programme combining human/porcine MDA and vaccination of pigs in Peru clearly demonstrated that elimination of taeniasis/cysticercosis at population level can be achieved [40]. Despite that, neurocysticercosis remains a major cause of acquired epilepsy and onset of seizure in developing countries [39].

Cystic and alveolar echinococcosis are prevalent in worldwide pastoral and rural communities, including medium-high income countries [41]. Apart from a few recent insights in the ultrasound-based prevalence study and disease fine-mapping analysis from large areas for cystic and alveolar echinococcosis, little advances were done for improving their clinical management, diagnostics, and benzimidazolic drugs which are only parasitostatic [4,42,43]. After the historical island-based elimination of cystic echinococcosis (Iceland, New Zealand, and Tasmania), and although a few decades ago a recombinant vaccine (Eg95) targeting the sheep intermediate host was developed, few gains have been obtained at continental level for its control [44]. To overcome the almost total absence of randomized controlled clinical trials, an international clinical register on cystic echinococcosis has been created, aiming at prospectively answer specific clinical questions for the management of patients [45].

## Ambitious targets for the WHO roadmap 2021–2030

Ninety percent reduction in the number of people in need of treatment against NTDs, 75% reduction in DALYs related to NTDs, 100 countries having eliminated at least 1 NTD and at least 2 NTDs eradicated in the world. These are the overarching impact-oriented global targets set by WHO in the road map for NTDs 2021–2030 to achieve the Sustainable Development Goals (SDGs) [11]. This new WHO roadmap was developed through an extensive global consultation with NTDs stakeholders that began in 2018 and culminated in the endorsement of the document by 194 Member States at the 73rd World Health Assembly in November 2020 [46].

WHO roadmap 2021–2030 also describes the integrated approaches needed to achieve these targets through cross-cutting activities built on 3 pillars: (1) accelerate actions aiming at reducing incidence, prevalence, morbidity, disability, and death due to NTDs by means of scientific advances, filling gap knowledge in research, providing new interventions and effective, standardized, and affordable diagnostics. (2) Intensify cross-cutting approaches by the integrated delivery of interventions that are common to several NTDs, mainstreaming them within national health systems in the context of universal health coverage, and enhancing coordination among stakeholders and related programmes such as WASH or vector control. Examples of these targets include: 75% reduction of deaths due to vector-borne NTDs, 75% MDA-integrated treatment coverage index, 40 countries adopting skin NTDs strategies, and 100% access to basic water supply, sanitation, and hygiene. (3) Change operating models and culture to facilitate countries to take ownership of their NTD programmes. Examples of these targets include: 90% of endemic countries, collecting and reporting data on NTDs disaggregated by gender.

In the new road map, each NTD is differently targeted for eradication (dracunculiasis and yaws), interruption of transmission (HAT–gambiense form, leprosy, onchocerciasis), elimination as a public health problem (Chagas disease, HAT–rhodesiense form, visceral leishmaniasis, lymphatic filariasis, rabies, schistosomiasis, STHs, trachoma), and control (Buruli ulcer, dengue and chikungunya, echinococcosis, foodborne trematode infections, cutaneous leishmaniasis, mycetoma, chromoblastomycosis and other deep mycoses, scabies and other ectoparasitoses, snake bite envenoming, taeniasis, and cysticercosis). Primary and secondary targets have been identified for each NTD [11].

## The future of NTD syndemics and the long wave of COVID-19

MDA remains a fundamental pillar for the concomitant control and elimination of NTD syndemics which should therefore have been managed as synergistic epidemics. Nevertheless, the dark side of these interventions relies on the potential onset of (particularly anthelmintic) drug resistance that in the future may jeopardize all these global efforts [47–49]. How to limit the environmental contamination of these drugs to avoid the insurgence of resistance is a relevant question, which has been too little debated in the scientific community. To circumvent this problem, new formulations and combination of more effective, soluble, age group-targeted drugs are in the pipeline (**Box 2**).

**Box 2. List of some new formulations and treatments of already existing drugs that have been recently developed and/or implemented against NTDs**

- Alternative regimen of ivermectin, diethylcarbamazine citrate, and albendazole to shorten the duration of required interventions targeting lymphatic filariasis MDA
- Fexinidazole for the oral treatment of HAT (first stage and nonsevere second stage of gambiense form)
- Paediatric formulation of mebendazole by chewable tablets for the treatment of STHs
- Paediatric formulation of praziquantel under development for the treatment of schistosomiasis
- New paediatric formulations of benznidazole and nifurtimox for Chagas disease
- Egaten (triclabendazole) for the treatment of human fascioliasis. Licensed by the US Food and Drug Administration (FDA)
- Antibiotic therapy (oral rifampicin plus clarithromycin extended release) to replace surgery for treatment of Buruli ulcer in some cases
- Azithromycin for treatment of yaws (instead of injectable benzylpenicillin in most circumstances)
- Clinical trials of fosravuconazole for the treatment of mycetoma
- Moxidectin approved by the FDA for the oral treatment of human onchocerciasis in patients aged 12 years and older
- Oxfendazole in Phase I (SAD and MAD) and Phase II (safety and efficacy) clinical studies for the human use against cysticercosis and trichuriasis
- Patent on new enantiopure and racemic anthelmintic formulations of soluble “salts of compounds having benzimidazolic structure” in preclinical studies

In parallel, global health efforts should focus on core preventive interventions such as new vaccines production aiming at the interruption of the transmission rather than curative interventions, and new diagnostic tools for early diagnosis and monitoring of drug resistance. Frontrunners vaccines for dengue, schistosomiasis, leishmaniasis, Chagas disease, and onchocerciasis are advancing in clinical trials, and some will be licensed soon [34,50–53]. WHO has also established an NTD Diagnostic Technical and Advisory Group (DTAG) to provide advice on diagnostics for control programmes and develop target product profiles for priority diagnostics [54]. All these to be integrated into future MDA campaigns.

Moreover, the ongoing coronavirus disease 2019 (COVID-19) pandemic in tropical and subtropical areas might jeopardize these developments on NTDs. The long wave of COVID-19 is having an impact not only in terms of coinfections between SARS-CoV-2 and all pathogens causing NTDs but also on delay or suspension of MDA and other community-based activities such as health facility services, control programmes, early diagnosis, drug supply, routine surveillance, and population-based surveys [55]. The main public health consequences of these disruptions due to COVID-19 might be identified as an increased mortality and morbidity associated to NTDs and delays in achieving the goals set for the 2021–2030 roadmap on NTDs. On top of all this, a challenging question was raised at the beginning of this pandemic, whether COVID-19 would be the next NTD [56]. According to “People’s Vaccine Alliance,” such question would be reasonable, since it has been estimated that 90% of people from low-income countries would not have access to vaccines against COVID-19 in 2021, while 14% of the world population represented by rich countries have already optioned 53% production of the more promising vaccines [57]. To overcome this vaccine nationalism, the COVAX partnership co-led by the triumvirate Vaccine Alliance (Gavi), the Coalition for Epidemic Preparedness Innovations (CEPI), and WHO is aiming at unbalancing this prediction, providing equitable access to vaccines at 2 billion people for the end of 2021 [58].

To conclude, WHO leads the way of this set of global interventions against NTDs, but it is up to Member States, donors, NGOs, academia, pharmaceutical and diagnostic companies, multilateral organizations, disease experts, implementing partners, and all other stakeholders to align their strategies under the "NTD brand" umbrella and take actions towards their prevention, control, elimination, and eradication. All these programmatic actions underpinning the end of the NTDs are based on the ethical principle that all lives have equal value. In this context, universal health coverage for the bottom billion affected by NTDs remains a global challenge for the years to come.
